# Tick Diversity and Abundance in Protected Natural Areas in Sicily, Southern Italy: A Baseline Ecological Study

**DOI:** 10.3390/ani16071081

**Published:** 2026-04-01

**Authors:** Ettore Napoli, Federico Cangialosi, Sergio Migliore, Paola Galluzzo, Elisa Maria Petta, Valeria Vaglica, Rosario Adragna, Davide Pepe, Francesca Gucciardi, Vincenza Cannella, Caterina Elen Culoma, Paulina Maria Lesiczka, Annalisa Guercio, Valeria Blanda

**Affiliations:** 1 Department of Veterinary Sciences, University of Messina, Viale Giovanni Palatuci 13, 98168 Messina, Italy; ettore.napoli@unime.it (E.N.); migliore.sergio@gmail.com (S.M.); 2 Istituto Zooprofilattico Sperimentale della Sicilia “A. Mirri”, Via G. Marinuzzi 3, 90129 Palermo, Italy; federico.cangialosi91@hotmail.com (F.C.); elisamariapetta@gmail.com (E.M.P.); vaglicavaleria@gmail.com (V.V.); adragna29@gmail.com (R.A.); davide.pepe@izssicilia.it (D.P.); francesca.gucciardi@izssicilia.it (F.G.); vincenza.cannella@izssicilia.it (V.C.); annalisa.guercio@izssicilia.it (A.G.); valeria.blanda@izssicilia.it (V.B.); 3 Center for Monitoring of Vectors, Netherlands Institute for Vectors, Invasive plants and Plant Health, Geertjesweg 15, 6706 EA Wageningen, The Netherlands; lesiczkapaulina@gmail.com

**Keywords:** ticks, Mediterranean, *Rhipicephalus bursa*, species richness, wild ungulates, seasonal dynamics

## Abstract

Ticks are vectors of pathogens affecting wildlife, livestock, and humans. Knowledge of their abundance, species composition, and ecological drivers is essential for an understanding of tick-borne disease ecology. In this study, four natural sites in Sicily (southern Italy) were selected, and 39 sampling events from April 2024 to August 2025 yielded 1200 ticks: 407 larvae, 474 nymphs, and 319 adults. Five genera were identified (*Dermacentor*, *Haemaphysalis*, *Rhipicephalus*, *Ixodes*, and *Hyalomma*); *Rhipicephalus bursa* was the most abundant species, followed by *Haemaphysalis punctata*. Most ticks were collected in spring–summer, except at one site where abundance peaked in autumn–winter. A significant association was found between site and season (*p* < 0.0001), but tick abundance per sampling event did not differ among sites (*p* = 0.431) or seasons (*p* = 0.769). No significant correlations emerged between tick abundance and environmental variants. However, significant associations were detected between site and sex (*p* < 0.0001), site and developmental stage (*p* < 0.0001), and species and developmental stage (*p* < 0.0001). Sites exclusively hosting wild boar showed higher species richness. These findings underscore the ecological complexity of tick populations in Mediterranean protected areas and provide baseline data useful for integrated tick surveillance and wildlife health management.

## Simple Summary

Tick populations are shaped by host availability, habitat, and microclimatic conditions. We conducted 39 sampling events across four natural areas in Sicily (southern Italy) to investigate tick abundance, species composition, and stage distribution. A total of 1200 ticks representing five genera were collected, with *Rhipicephalus bursa* being the most abundant species. Larvae and nymphs predominated, except in one site where adults were more common. Tick abundance did not vary significantly among sites or seasons, and no consistent relationship with saturation deficit was detected. Ungulate community composition influenced tick species richness, with sites dominated by wild boar exhibiting higher richness. These findings suggest that tick populations maintain relatively stable abundance across sites and seasons, with a predominance of immature stages, while host community composition could influence tick species richness in Mediterranean habitats. Overall, this study provides a baseline for future monitoring and risk assessment related to tick-borne diseases in the Mediterranean region.

## 1. Introduction

Hard ticks (Ixodidae) are listed among the main important vectors of arthropod-borne pathogens in Europe [1,2,3]. The epidemiology of arthropod-borne pathogens is strongly influenced by the presence and the distribution of the vectors, which are in turn modulated by the presence of suitable hosts. Therefore, studying the phenology and ecology of the vectors is mandatory for better comprehension of the epidemiology of tick-borne diseases (TBDs).

Ticks typically inhabit specific ecological niches that provide favorable microclimatic conditions [4]. While weather conditions may affect tick distribution, host-seeking behavior, and pathogen transmission dynamics [5], the phenology of ticks is above all influenced by the presence and abundance of animal hosts [6]. In Mediterranean ecosystems, tick phenology is primarily shaped by the presence of livestock, wild ungulates, and mesocarnivores.

The Italian tick fauna is composed of more than 40 species [5,7,8]; moreover, the Italian peninsula has been recognized as a potential hybridization site of some *Ixodes* species [9].

In Sicily, the animals whose population dynamics have changed significantly over the last two decades are the wild boar (*Sus scrofa*) and the fallow deer (*Dama dama*) [10,11,12]. The population growth of wild boar and fallow deer, combined with insufficient surveillance strategies, poses a serious threat to both human and animal health. These animals can act as maintenance hosts for several endemic tick species and reservoirs for tick-borne pathogens [13]. Thanks to their extraordinary adaptability and opportunistic feeding habits, wild boar and fallow deer frequently share natural resources with livestock and humans, thereby enhancing the risk of interspecies pathogen transmission [14]. Indeed, wild ungulates may serve as hosts for *Dermacentor marginatus*, *Dermacentor reticulatus*, *Hyalomma lusitanicum* and *Ixodes ricinus* [15] and may contribute to the circulation of zoonotic diseases such as Lyme disease and tick-borne encephalitis [16]. Notably, a high abundance of ticks and tick-borne agents has been reported in wild boar hunting areas of southern Italy with a relevant zoonotic risk for wild boar hunters and hunting dogs [17].

However, most studies on ticks, in Italy have concentrated on specimens collected directly on the host and on the specific interaction between tick and host [17,18,19], while investigations into the ecology of free-living (off-host) ticks remain comparatively limited [5,9,20,21]. Focusing solely on ticks collected from hosts can lead to biased estimates of prevalence, as such approaches do not capture the full off-host population. Integrating both on-host and off-host surveillance using standardized methods provides a more comprehensive picture of pathogen circulation and transmission dynamics [6]. Furthermore, studying on-host tick populations, particularly in wildlife, often relies solely on passive surveillance. Therefore, for a holistic understanding of tick phenology and the eco-epidemiology of the related tick-borne pathogens (TBPs), active monitoring of host-seeking ticks is essential, especially in protected woodlands where humans, livestock, and wildlife coexist, increasing the risk of tick-borne disease (TBD) transmission/circulation.

Sicily, the largest island of the Mediterranean Basin (ca. 25,711 km^2^), presents a wide ecological and geological diversity. Its territory exhibits marked geomorphological diversity, ranging from the northern mountain chains (Peloritani, Nebrodi, and Madonie) to southern plateaus and coastal plains. The Mediterranean climate, characterized by hot, dry summers and mild, humid winters, supports heterogeneous biotopes that favor the persistence of free-living tick populations. The Sicilian territory includes several protected areas, covering approximately 11% of the total surface area. These areas comprise five regional parks, 76 regional nature reserves, six marine reserves, and several other natural areas. In these protected areas, wild and domestic animals, humans, and tick populations coexist, enhancing the risk of TBPs transmission.

Over the last decade, a significant increase in the populations of fallow deer and wild boar has been observed in Sicily, particularly within protected natural areas. Despite this evident expansion, systematic population censuses have not been conducted. Currently, official data are limited to records of presence–absence of fallow deer and/or wild boar, while reliable information on their numerical abundance and population density is lacking. It should be noted that the sampling areas are designated protected natural areas where livestock grazing is not permitted; consequently, the presence of domestic animals is virtually absent. However, wild ungulates can freely move outside these areas and may therefore act as carriers, dispersing ticks beyond the boundaries of the protected sites.

Despite Sicily’s strategic geographic position in the heart of the Mediterranean Basin and its role as a natural bridge between Europe and North Africa, data on tick ecology in this region remain limited, outdated, and fragmentary. Sicily lies along major Afro-Palearctic migratory flyways, serving as an important stopover site where millions of birds rest during their long-distance movements between Africa and Europe. This seasonal migratory flow is not merely an ornithological event but represents a systemic mechanism for the large-scale transport of parasites. Historical evidence has long established that northward-bound migratory birds are responsible for the transcontinental transport of African ticks, particularly those of the genus *Hyalomma*, whose immature stages, particularly nymphal ones [22], remain attached to the host long enough to overcome significant geographical barriers [23]. A recent comprehensive review of the European literature identified over 50 tick species associated with nearly 300 avian species, especially "hitchhikers” belonging to specific ground-foraging families [24], confirming that birds are the most significant long-distance dispersers of ixodid ticks across the continent [25,26]. However, the effectiveness of this transport is not random; it is closely tied to the ecology of the species involved. Upon reaching territories like Sicily, these ticks do not merely act as passive travelers but attempt to integrate into local food webs.

The current climate change scenario acts as a catalyst in this process, making the Sicilian environment increasingly thermally suitable for the permanent establishment of exotic tick populations. Moreover, considering the high environmental variability of Sicily, monitoring of the different arthropod habitat could offer insights relevant to the wider Mediterranean basin. This lack of knowledge may limit the understanding of local transmission dynamics and may obscure the potential for introduction and establishment of novel tick species and the related tick-borne pathogens in the central Mediterranean area.

The present study therefore aims to characterize the ixodid fauna selected in four protected woodlands in Italy (Southern Europe), assess the role of biotic and abiotic variables in the tick species populations, and evaluate the influence of the presence of different wild ungulate populations on tick distribution and abundance.

## 2. Materials and Methods

### 2.1. Study Area

Four natural sites in Sicily (protected areas) were selected a priori based on an integrated assessment of key ecological, wildlife, and anthropogenic variables. In particular, the main selection criteria included Mediterranean climatic–ecological conditions favorable to tick survival, the expansion of wild ungulate populations influencing host availability, and indicators of tick and host presence. Finally, the proximity of human settlements, recreational areas, and agro-pastoral activities was evaluated to account for increased human–animal–environment interface.

**Figure 1:**
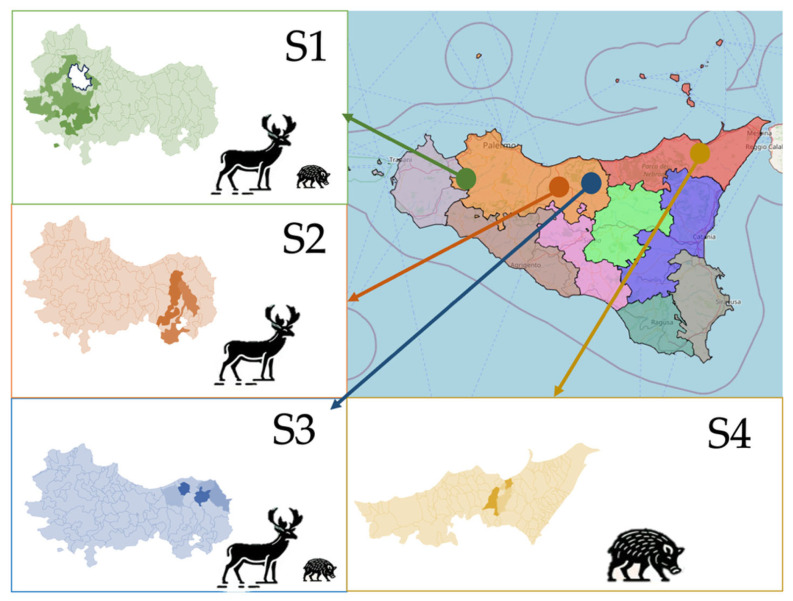
Schematic map showing the selected study sites.

Each sampling event was consistently conducted in the same location, as defined by the coordinates reported below in the specific description of the different study sites. In Figure 1 is reported a map with the selected study sites, with brief descriptions below.

Site 1 (S1): The Ficuzza Nature Reserve (37°52′29.5″ N, 13°22′41.7″ E) in Province of Palermo, western Sicily, spans 7397 ha of forests, rocky slopes, and wetlands, 600–1613 m a.s.l., with a Mediterranean montane climate characterized by cool, rainy winters (annual rainfall ≈ 900–1200 mm) and warm, dry summers moderated by altitude, resulting in frequent mists and occasional snow on Rocca Busambra. The area is rich in biodiversity and endemic species (e.g., pine marten, *Martes*; porcupine, *Hystrix cristata*; weasel, *Mustela nivalis*; red fox, *Vulpes*; European wild rabbit, *Oryctolgaus cuniculus*; and small rodents). Fallow deer and wild boar populations are widely present, representing a serious problem for the Ficuzza reserve management.

The Madonie Regional Natural Park (37°87′ N, 14°05′ E), located in Province of Palermo, covers approximately 39,900 ha and spans an altitudinal range from 200 to 1979 m a.s.l. The park is characterized by a Mediterranean to montane climate, with mild, wet winters (annual precipitation of approximately 800–1400 mm) and dry, temperate summers. At higher elevations, temperatures are cooler, with frequent fog and winter snowfall on peaks such as Pizzo Carbonara. Owing to its geographical extent, the Madonie Regional Natural Park is the third largest natural park in Sicily and encompasses a wide variety of ecotypes; therefore, two study sites were selected within the park.

Site 2 (S2): Madonie Alte (37°54′01.1″ N, 13°59′43.0″ E), a wooded area in the municipality of Petralia sottana, Petralia soprana, Polizzi Generosa, ranging from 920 to 1147 m a.s.l.; the area is characterized by the presence of a large population of fallow deer.

Site 3 (S3): Madonie Basse (37°55′16.4″ N, 13°56′36.9″ E), a rural area, with pasture for livestock and cultivated fields in the municipality of San Mauro Castelverde, Collesano, ranging from 560 to 700 m a.s.l. The study area hosts abundant populations of fallow deer and wild boar. In this area, wild ungulates commonly co-occur with domestic cattle on shared pastures.

Site 4 (S4): The Altiplano dell’Argimusco (37°59′20.2″ N, 15°04′04.7″ E) in the Province of Messina spans a high plateau at ~1165–1230 m a.s.l., with a mountain-influenced Mediterranean climate: mild, wet winters and cool, relatively dry summers; altitude moderates extremes, and frequent mists may form. The area supports wildlife including foxes, martens, weasels, wild boar, and birds of prey.

### 2.2. Meteorological Data

Meteorological variables were obtained from local weather stations of the *Servizio Informativo Agrometeorologico Siciliano* (SIAS) located in the vicinity of the four sampling sites. The stations considered were Monreale Bifarera (663 m from S1), Polizzi Generosa (2220 m from S2 and 9590 m from S3), and Montalbano Elicona (6560 m from S4). To monitor climatic parameters over the long term, these data were used. The meteorological variables considered were the temperature (°C), the relative humidity (%), and the rainfall (mm). Temperature and maximum relative humidity were calculated as the average values over the 10 days preceding each sampling date, while precipitation was expressed as the cumulative total over the same period. Moreover, during the sampling days, a battery-powered portable thermo-hygrometer was deployed. Short-term averages over the 10 days preceding sampling were combined with on-site measurements to capture both cumulative and instantaneous microclimatic conditions influencing tick activity, survival, and development. This approach reflects the recent climatic history shaping tick abundance and distribution at each site. Rainfall was expressed as the cumulative precipitation (mm) over the previous 10-day period. Moreover, the saturation deficit (SD), an index characterized by the difference between the saturation relative humidity and the actual relative humidity of a volume of air, was calculated as follows: SD = (1 − RH/100) × 4.9463 × e^0.0621×T^, where RH is relative humidity and T is temperature [27]. In addition, during each sampling session, operators carried a portable digital thermo-hygrometer to verify whether the meteorological parameters recorded in the field differed significantly from those reported by the reference SIAS weather station.

### 2.3. Tick Collection and Identification

Selected study areas were monitored from April 2024 to August 2025, and sampling was conducted according to planned temporal intervals reflecting seasonal tick activity. The sampling activity was carried out between 11:00 a.m. and 2:00 p.m. Each sampling session lasted 30 ± 5 min per site and was conducted simultaneously by two operators using dragging and flagging techniques with cotton flannels measuring 100 cm × 115 cm and 100 cm × 100 cm. Collected ticks were removed with forceps and stored in plastic vials labelled with the collection date and site. Once received by the laboratory, samples were stored at −80 °C to ensure they remained well-preserved for potential subsequent molecular analyses. Specimens were identified morphologically using standard dichotomous keys [3,7,28]. Adult ticks were identified based on diagnostic morphological characters, including scutum ornamentation and punctuation, basis capituli morphology, presence or absence of festoons, spiracular plate morphology, coxal spur configuration, adanal and accessory plates (in males), and genital aperture position [3,7,29]. Nymphs and larvae were identified using stage-specific morphological criteria, including scutal shape and dimensions, chaetotaxy, hypostomal dentition formula, basis capituli morphology, palpal segment proportions, and leg segmentation patterns, according to updated taxonomic keys and morphological atlases [3,30,31,32]. Specimens were thawed at room temperature prior to examination. No molecular analyses were performed within the scope of the present study.

### 2.4. Statistical Analysis

Tick abundance was defined as the total number of ticks collected in each sampling event. Species richness was calculated as the total number of species identified at each study site, considering all sampling events carried out at that site. Species diversity was computed as the total number of species for each sampling event conducted at each study site.

Based on the month of sampling, the variable “Season” was defined, comprising two categories: ‘Spring–Summer’ (April–September) and ‘Autumn–Winter’ (October–March) in order to assess overall seasonal differences in tick abundance and species composition. Categorical variables were studied using Chi-squared tests, while Analysis of Variance (ANOVA), Kruskal–Wallis tests or Welch’s, as appropriate, were used to evaluate the differences among groups [33].

Prior to the data analysis, data normality was assessed using the Shapiro–Wilk test. When data were not normally distributed, logarithmic transformations were applied [log_10_(x + 1)], and the homogeneity of variances among groups was evaluated using Bartlett’s test. Multiple tests were corrected using the Bonferroni method.

The influence on tick richness and abundance of the presence of the different wild ungulate populations (i.e., wild boar + fallow deer, fallow deer only, or wild boar only) was calculated using ANOVA or Kruskal–Wallis tests.

Shannon’s index (*H*), Gini Diversity index (*I*) and Pielou’s evenness index (*R*) were calculated for each study site according to Kunakh and collaborators [34].

In particular, the Gini index (*I*) was calculated following the formula:

(1) $$I\;=\;1\;-\;\;\sum_{j\;=\;1}^{p}f_{j}^{2}$$

where *p* is the number of observed species and f_j_ is the proportion of ticks belonging to species *j*. The index *I* was normalized by dividing it by $\frac{p\;-\;1}{p}$, thereby obtaining a standardized measure ranging from 0 to 1. Higher values reflect greater heterogeneity, indicating a more even distribution of ticks among the observed species.

Shannon’s index (H′) was calculated using the formula:

(2) $$H^{\prime }\;=\;-\sum_{j\;=\;1}^{p}\frac{n_{j}}{N}\;\ln\frac{n_{j}}{N}$$

where *p* is the number of observed species, *nj* = number of ticks belonging to a single species *j*, and *N* = total number of ticks. The higher the index, the more evenly ticks are distributed among the observed species.

The Pielou index (*R*) was evaluated as follows:

(3) $$R\;=\;\frac{H\prime }{\ln\left(p\right)}$$

In each formula, ln(*p*) represents the theoretical maximum of *H*′, and *R* is the normalized version of *H*′, ranging from 0 to 1; the higher the R value, the more evenly ticks are distributed among the observed species.

Correlations between tick abundance and climatic variables (i.e., temperature, humidity, rainfall, and saturation deficit) were assessed using Pearson’s r or Spearman’s rs as appropriate, for each site and season. The analysis was conducted within the R environment (R CoreTeam 2025 4.5.1, R Foundation for Statistical Computing, Vienna, Austria) [35], with significance at *p* < 0.05.

## 3. Results

A total of 39 sampling events were conducted across the study sites. Table 1 summarizes the mean, standard deviation, and coefficient of variation (CV) of tick abundance per sampling event for each site.

Tick count distributions deviated significantly from normality at all sites, except S1 (Shapiro–Wilk test, p = 0.563). After logarithmic transformation, no significant departures from normality were detected (Table 1). Homogeneity of variances was assessed using Bartlett’s test, which revealed significant heterogeneity among sites (K = 9.642, *p* = 0.022). Consequently, Welch’s ANOVA—robust to unequal variances—was applied in place of classical ANOVA. The Welch test indicated no significant differences in mean tick abundance among sites (F = 0.995, *p* = 0.431), suggesting comparable tick numbers per sampling event across the study area.

The meteorological data recorded by the reference weather stations on the sampling days were compared with those measured on the same dates using the portable thermo-hygrometer, and no significant differences were detected that could have influenced the results.

In Table 2 is summarized the correlations between tick abundance and the environmental parameters. For each parameter, no significant patterns were detected, although some trends were observed in some instances.

With respect to the correlations between tick abundance and mean temperature (Table 3), both the magnitude and direction of the correlations varied (e.g., r = –0.554 in S1; r = 0.398 in S2; r = 0.009 in S4), but no consistent pattern was observed.

The correlation between tick abundance and the mean of the relative humidity maximum was positive in S1 (Table 3) but not significant according to the *p*-value of the test (r = 0.531, *p* = 0.372).

Regarding the correlation between tick abundance and total precipitation in the 10 days prior to sampling, although none of the estimates were significant (Table 3), a negative correlation could be observed at site S2 (r = −0.398, *p* = 1); instead, at S3, which also belongs to the Madonie Park, the correlation appeared positive (r = 0.284, *p* = 1). Regarding the Saturation deficit, in S1, the correlation between tick abundance and the mean saturation deficit was −0.554; in S2, the same measure was 0.398; and in S4, it was 0.009 (Table 3). There may have been differences between the correlations measured in each study site, but all of the estimated values were not statistically significant.

Based on the month of sampling, the variable “Season” was defined, comprising two categories: ‘Spring–Summer’ (April–September) and ‘Autumn–Winter’ (October–March). Seasonally, more ticks were collected during Spring–Summer (n = 738) than during Autumn–Winter (n = 462) (Table 3). However, the distribution of tick abundance per sampling event did not meet normality assumptions, and logarithmic transformation failed to normalize the data (Shapiro–Wilk test, *p* < 0.05 for both seasons). Given these violations, non-parametric methods were applied. Variances in tick abundance per sampling event were homogeneous across seasons (Bartlett’s test: K = 0.099, *p* = 0.753), allowing the use of the Kruskal–Wallis test, which revealed that tick abundance per sampling event did not differ significantly between seasons (KW = 0.086, *p* = 0.769). In all sites except S4, most ticks were collected in Spring–Summer; in S4, the highest number occurred in Autumn–Winter. A Chi-squared test indicated a significant association between site and season (X^2^ = 261.799, *p* < 0.0001).

A total of 1200 ticks were collected and identified, consisting of 407 larvae, 474 nymphs, and 319 adults (163 males and 156 females) (Table 4). The highest counts were recorded at S4, followed by S3 and S1. Juvenile stages predominated at S2 and S4 (92% and 83%, respectively), whereas at S3, adult males and females were proportionally more abundant. Unlike the other sites, at S3, the number of adults equaled the number of nymphs. Chi-squared analyses indicated significant associations between study site and sex (X^2^ = 174.537, *p* < 0.0001), and between study site and developmental stage (X^2^ = 420.729, *p* < 0.0001).

Across the four sites, ticks belonged to five genera: *Dermacentor*, *Haemaphysalis*, *Rhipicephalus*, *Ixodes*, and *Hyalomma* (Table 5, Table S1). *Rhipicephalus bursa* was the most frequently collected species overall (except in S1), followed by *Haemaphysalis punctata* (predominantly at S4 and S3), *Ixodes ricinus* (mainly in S1), and *D. marginatus* (mostly in S2 and S3). A single *Hyalomma lusitanicum* female specimen was collected at S1. Consistent with total counts, most individuals across species were larvae or nymphs. A significant association emerged between tick species and developmental stage (X^2^ = 406.641, *p* < 0.0001).

As shown in Table 5 and Table 6, most of the ticks collected belonged to the species *Rhipicephalus bursa,* with 523 individuals identified as juveniles: 377 larvae and 146 nymphs. Even for the other species, most ticks were larvae or nymphs (Table 6). The Chi-squared tests underlined an association between the variable species and stages ($X^{2}$ = 406.641, *p* < 0.0001).

Sampling sites were grouped according to the presence of wild ungulates into three categories: “*Sus scrofa*” (site S4), “*Fallow deer*” (site S2), and “Both” (sites S1 and S3). Tick abundance per sampling site was initially found to be non-normally distributed across these groups; however, log-transformed values conformed to normality, allowing application of classical ANOVA, which detected no differences among ungulate groups (F = 0.598, *p* = 0.555). Conversely, the number of species per sampling event showed a non-normal distribution, prompting use of the Kruskal–Wallis test. Significant differences in species richness among ungulate groups were detected (KW = 8.447, *p* = 0.015). Pairwise Wilcoxon comparisons highlighted a single significant contrast: “*Sus scrofa*—Both” (*p* = 0.012), suggesting that sites in which, among the wild ungulates, only the presence of wild boar was registered may harbor a higher tick species richness (“*Sus scrofa*”: 2.38 species identified per sampling, “Both”: 1.50).

The different diversity indexes calculated are summarized in Table 7. The normalized Gini index (*I* ∈ [0;1]) for S2 (I = 0.412) indicates strong dominance by a single species, consistent with the species composition displayed in Table 5. Although S3 and S4 share the same Shannon’s index (*H* = 0.883), S4 exhibits a lower Gini index (*I* = 0 0.664), reflecting higher richness, but most of the ticks collected may belong to the same species. Conversely S3 has a higher Gini index (*I* = 0.771) because ticks are more evenly distributed across the three species collected.

## 4. Discussion

The present study provides updated data regarding the ixodid fauna dynamics and composition in four protected woodland areas in Sicily, Italy (Southern Europe), utilizing standardized active surveillance methods. Our overall collection of 1200 ticks (consisting of 407 larvae, 474 nymphs, and 319 adults) confirms that these Mediterranean woodlands sustain significant populations of host-seeking ticks. Overall, the results are consistent with general ecological patterns reported for European ixodid ticks, and additionally highlight the influence of the specific characteristics of the sampling sites considered in this study. This active, standardized approach addresses the recognized limitation of relying solely on passive, on-host surveillance methods, providing a more comprehensive understanding of off-host tick ecology [6].

Five genera were identified *Rhipicephalus*, *Haemaphysalis*, *Dermacentor*, *Ixodes*, and *Hyalomma*, with *Rhipicephalus bursa* being the most abundant species at most sites. As reported by other studies [5,7], *R. bursa* and *Haemaphysalis punctata* commonly dominate the Italian tick fauna, due to their strong association with livestock and wild ungulates [5]. These results align with the landmark studies [36,37] conducted on Croatian islands (Cres, Lošinj, and Brač), which identified a comparable species assemblage and emphasized the dominance of *Rhipicephalus* and *Haemaphysalis* in insular and coastal environments. Specifically, the high prevalence of *R. bursa* observed in Sicily mirrors the observations on the island of Brač. In both cases, xerothermic conditions appear to favor this genus over *Ixodes ricinus*, the latter remaining restricted to more humid microclimates.

This predominance of Mediterranean-adapted taxa is consistent with broader European surveys, which highlight the role of local climatic regimes in shaping regional tick communities [2,38].

Sicily’s location and habitat diversity make it a potential hotspot for tick dispersal into Europe. Migratory birds can transport immature ticks over long distances, promoting the introduction of exotic species or the northward expansion of thermophilic taxa such as *Hyalomma* spp. [1,39]. The sporadic detection of *H. lusitanicum* in this study likely reflects such dispersal events.

These findings highlight the island’s role in continental tick dynamics and underscore the importance of ongoing surveillance to monitor emerging tick-borne pathogen risks. Marked differences in species dominance were detected among sites, particularly in S1 or S2, where a single species accounted for most individuals, as reflected by the lower normalized Gini index.

The presence of *I. ricinus* and *D. marginatus* is of particular interest, given their role as principal vectors for highly relevant zoonotic pathogens, including *B. burgdorferi* s.l., an agent of Lyme disease and tick-borne encephalitis virus, in the wider Palearctic context [15].

Across sites, immature stages (larvae and nymphs) predominated, except at S3, where adults were relatively more abundant, and their number equaled that of nymphs. A dominance of larvae and nymphs in drag/flag samples is widely reported, as larvae and nymphs are both more abundant numerically and more likely to quest within the vegetation layer accessible to sampling [1]. However, sites with abundant large ungulates may yield proportionally more adults, given that adult *Rhipicephalus* and *Haemaphysalis* rely on medium-to-large mammals for feeding [38].

Although a higher number of ticks was collected during Spring–Summer compared to Autumn–Winter, no significant seasonal differences in tick abundance per sampling event were observed. This general pattern is consistent with the established seasonal activity peaks of most hard ticks in the Mediterranean region, which are driven primarily by favorable temperature and humidity conditions [5].

Many European studies report clear seasonal peaks in nymphal activity, particularly for *I. ricinus,* during spring and early summer [1]. It has long been known that the persistence of tick populations relies heavily on the availability of small hosts, such as reptiles for immature stages and wild ungulates for adults, suggesting that local vegetation structure and wildlife composition are the primary drivers of population density [40].

However, Mediterranean tick species, such as *Rhipicephalus* spp. and *Hae. punctata*, may quest year-round or exhibit extended activity periods depending on temperature and humidity conditions [5], resulting in a seasonal contrast attenuation.

The heterogeneity observed across sites (e.g., S4 recording the highest number collected in Autumn–Winter) suggests an influence of site-specific characteristics and host availability [4,41].

The atypical peak in S4 during Autumn–Winter supports this site-specific interpretation and mirrors observations from Mediterranean regions where certain species show late-season activity in cooler, more humid months [2].

Correlations between tick abundance and saturation deficit were weak and inconsistent across sites. While saturation deficit is a well-known driver of questing behavior for desiccation-sensitive ticks such as *I. ricinus* [1], studies have repeatedly emphasized that macro-scale climatic indicators may fail to capture microclimatic refugia that strongly buffer ticks from desiccation [41]. The lack of a clear relationship in this study therefore aligns with the growing understanding that microhabitat conditions, vegetation cover, and soil moisture can influence the short-term questing activity of ticks.

Tick abundance per sampling event did not significantly differ among sites characterized by the presence of different wild ungulate groups. However, species richness did, with a significant contrast between areas characterized by wild boar presence only and those where both wild boars and fallow deer were present. Remarkably, the higher proportion of adults in sites S1 and S3, hosting both wild boar and fallow deer, is consistent with the presence and activity of large ungulate hosts that have experienced substantial changes in population and distribution across Sicily over the past two decades [9,10,11]. This supports the hypothesis that host identity influences tick community structure more strongly than overall tick density. As pointed out by several authors, wild ungulates are essential hosts for adult ticks, facilitating their reproductive cycle; however, ungulate species differ in grooming behavior, body size, habitat use, and suitability for specific tick taxa, all of which may drive differences in species composition even when overall tick productivity remains comparable [15,38]. The demographic increase and the opportunistic feeding habits of these ungulates enhance the risk of interspecies pathogen transmission to livestock and humans [14].

In the Altiplano dell’Argimusco (S4), where only wild board harbored, a higher tick richness per collection event could suggest that wild boar may promote or limit certain species. Previous studies in southern Italy have similarly demonstrated that habitat-specific host communities lead to significant spatial variation in tick diversity [5].

Despite the standardized sampling design, fine-scale spatial heterogeneity may still have influenced tick abundance and species composition. Microtopographic features, vegetation structure, host activity patterns, and small-scale variations in soil moisture can generate localized ecological conditions that are not fully captured by site-level analyses. In addition, meteorological data were obtained from stations located between 650 m and 10 km from the sampling sites. Although these distances were explicitly reported and acknowledged as a limitation, macroclimatic measurements may not accurately represent the microclimatic conditions experienced by ticks in their immediate habitat. In Mediterranean environments, local topography and vegetation can strongly modulate temperature and humidity near the ground, potentially obscuring climate–abundance relationships. Furthermore, correlations were calculated between tick abundance per sampling event and meteorological variables averaged over the 10 days preceding sampling. While this approach was intended to account for short-term climatic influences on tick activity, it may not adequately capture delayed or cumulative effects of environmental conditions on development, survival, and host-seeking behavior.

Accordingly, the absence of significant correlations between tick abundance and environmental variables should be interpreted within the context of these methodological considerations; nevertheless, the present findings provide a useful baseline for future investigations conducted at finer spatial and temporal scales [42].

Therefore, our results confirm that climatic factors are not the only determinants of tick distribution and abundance, but that these are also determined by a complex interaction of ecological and environmental factors, including host availability and density, habitat fragmentation, biodiversity loss, and human activities.

## 5. Conclusions

In conclusion, despite local variation in species composition and stage distributions, overall tick abundance remained comparable among the four studied sites. No significant seasonal differences in tick abundance per sampling event were detected. The dominance of *Rh. bursa* and *Hae. punctata*, together with the sporadic detection of *Hy. lusitanicum*, reflects a tick community typical of Mediterranean ecosystems, suggesting the influence of regional climatic conditions and host availability in shaping local tick populations.

Taken together, these findings emphasize the ecological complexity underlying tick populations in Mediterranean environments. They underscore the need for integrated approaches that consider microclimatic conditions, fine-scale habitat structure, and host community composition to better understand tick distribution patterns and potential shifts under ongoing environmental change. Future research incorporating microclimate measurements, multi-year sampling, and host-focused ecological metrics may provide a more comprehensive understanding of the mechanisms shaping tick communities in these systems.

## Figures and Tables

**Table 1:** Number of sampling occasions (N Ev.) and number (N) of tick collected, Mean, Standard Deviation (St. Dev.) and Coefficient of variation (CV) in the different study sites. W = Shapiro–Wilk result; W.log = SW statistic of transformed data.

Site	Geographical Coordinates		N Ev.	N	Mean	St. Dev.	CV	W	*p*-Value	W.log	*p-value*.log
	N	E									
S1	37°52′29.5″	13°22′41.7″	12	164	13.67	5.51	0.40	0.945	0.563	0.923	0.311
S2	37°54′01.1″	13°59′43.0″	4	131	32.75	31.56	0.96	0.724	0.022	0.832	0.174
S3	37°55′16.4″	13°56′36.9″	10	280	28.00	22.97	0.82	0.841	0.045	0.878	0.123
S4	37°59′20.2″	15°04′04.7″	13	625	48.08	73.31	1.52	0.630	0.000	0.905	0.154

**Table 2:** Correlations between the environmental parameters and tick abundance.

	Mean Temperature			Relative Humidity			Rainfall Over the Previous 10-day Period			Saturation Deficit		
Site	Rho	*p*.value	p.bon	Rho	*p*.value	p.bon	Rho	*p*.value	p.bon	Rho	*p*.value	p.bon
**S1**	0.531	0.093	0.372	0.314	0.347	1.000	0.314	0.347	1.000	−0.554	0.077	0.308
**S2**	−0.398	0.602	1.000	−0.398	0.602	1.000	−0.398	0.602	1.000	0.398	0.602	1.000
**S3**	−0.167	0.645	1.000	0.284	0.426	1.000	0.284	0.426	1.000	−0.119	0.744	1.000
**S4**	0.099	0.747	1.000	−0.382	0.198	0.792	−0.382	0.198	0.792	0.009	0.976	1.000

**Table 3:** Number of ticks collected in Autumn–Winter (October–March) and Spring–Summer (April–September) in the four study sites.

	Season		
Site	Autumn–Winter	Spring–Summer	Total
S1	86	78	164
S2	0	130	130
S3	30	248	278
S4	349	279	628
Total	462	738	1200

**Table 4:** Number and stage of ticks collected according to study site.

Stage						
Site	Larvae	Nymph	Total adult	Female	Male	Total
S1	3	96	65	32	33	164
S2	108	11	11	7	4	130
S3	12	131	135	42	93	278
S4	290	230	108	75	33	628
Total	413	468	319	156	163	1200

**Table 5:** Species and number of ticks collected across study sites over the sampling period.

Species						
Site	*Rhipicephalus bursa*	*Haemaphysalis punctata*	*Ixodes ricinus*	*Dermacentor marginatus*	*Hyalomma lusitanicum*	*Total*
**S1**	41	2	117	3	1	164
**S2**	106	2	2	20	0	130
**S3**	180	39	59	0	0	278
**S4**	411	168	31	18	0	628
**Total**	**738**	**211**	**209**	**41**	**1**	**1200**

**Table 6:** Species and stages of ticks collected in the study period.

	Adult			Larvae	Nymph	Total
Species	N. Adults	Female	Male			
*Rhipicephalus bursa*	235	104	131	357	146	738
*Haemaphysalis punctata*	23	12	11	29	159	211
*Ixodes ricinus*	43	27	16	7	159	209
*Dermacentor marginatus*	17	12	5	20	4	41
*Hyalomma lusitanicum*	1	1	0	0	0	1
Total	319	156	163	413	468	1200

**Table 7:** Diversity indices and richness according to study site.

Parameters	S1	S2	S3	S4
Pielou’s evenness index (*R*)	0.46	0.42	0.80	0.64
Gini diversity index (*I*)	0.54	0.41	0.77	0.66
Shannon’s index (*H*′)	0.75	0.58	0.88	0.88
Richness	5	4	3	4

## Data Availability

The original contributions presented in this study are included in the article/Supplementary Material. Further inquiries can be directed to the corresponding author.

## Supplementary Materials

The following supporting information can be downloaded at: https://www.mdpi.com/article/10.3390/ani16071081/s1, Table S1: Tick species and developmental stages in the four studied sites. M: Male; F: Female; N: Nymph; L: Larvae.
