# Supplementary material for: Tick Diversity and Abundance in Protected Natural Areas in Sicily, Southern Italy: A Baseline Ecological Study
Source: Animals (Basel). 2026 Apr 1;16(7):1081. doi: 10.3390/ani16071081 (PMC13072420; doi:10.3390/ani16071081)
Supplement: Supplementary file 1 [file animals-16-01081-s001.zip › animals-4146983-supplementary.pdf]

Table S1. Tick species and developmental stages in the four studied sites. M: Male; F: Female; N: Nymph; L: Larvae

	Ixodes ricinus					*Rhipicephalus bursa*					*Dermacentor marginatus*					*Haemaphysalis punctata*					*Hyalomma lusitanicum*				
**Site**	Tot	M	F	N	L	Tot	M	F	N	L	Tot	M	F	N	L	Tot	M	F	N	L	Tot	M	F	N	L
**S1**	117	12	13	89	3	41	19	15	7	0	3	2	1	0	0	2	0	2	0	0	1	0	1	0	0
**S2**	2	0	0	0	2	106	4	7	9	86	20	0	0	0	20	2	0	0	2	0	0	0	0	0	0
**S3**	59	2	3	54	0	180	80	39	50	11	0	0	0	0	0	39	11	0	27	1	0	0	0	0	0
**S4**	31	2	11	16	2	411	28	43	80	260	18	3	11	4	0	168	0	10	130	28	0	0	0	0	0
**Total**	**209**	**16**	**27**	**159**	**7**	**738**	**131**	**104**	**146**	**357**	**41**	**5**	**12**	**4**	**20**	**211**	**11**	**12**	**159**	**29**	**1**	**0**	**1**	**0**	**0**
